# Position-dependent hearing in three species of bushcrickets (Tettigoniidae, Orthoptera)

**DOI:** 10.1098/rsos.140473

**Published:** 2015-06-09

**Authors:** Reinhard Lakes-Harlan, Jan Scherberich

**Affiliations:** Institute for Animal Physiology, AG Integrative Sensory Physiology, Justus-Liebig- Universität Gießen, Heinrich-Buff-Ring 26, Gießen 35392, Germany

**Keywords:** auditory system, hearing threshold, frequency tuning, evolution of sensory system

## Abstract

A primary task of auditory systems is the localization of sound sources in space. Sound source localization in azimuth is usually based on temporal or intensity differences of sounds between the bilaterally arranged ears. In mammals, localization in elevation is possible by transfer functions at the ear, especially the pinnae. Although insects are able to locate sound sources, little attention is given to the mechanisms of acoustic orientation to elevated positions. Here we comparatively analyse the peripheral hearing thresholds of three species of bushcrickets in respect to sound source positions in space. The hearing thresholds across frequencies depend on the location of a sound source in the three-dimensional hearing space in front of the animal. Thresholds differ for different azimuthal positions and for different positions in elevation. This position-dependent frequency tuning is species specific. Largest differences in thresholds between positions are found in *Ancylecha fenestrata*. Correspondingly, *A. fenestrata* has a rather complex ear morphology including cuticular folds covering the anterior tympanal membrane. The position-dependent tuning might contribute to sound source localization in the habitats. Acoustic orientation might be a selective factor for the evolution of morphological structures at the bushcricket ear and, speculatively, even for frequency fractioning in the ear.

## 1. Background

Sensory organs capture physical characteristics of the environment and allow orientation in the habitat. For orientation, directional cues of sensory modalities are of pivotal importance. Sound waves are highly directional, although reflections and diffraction reduce their usage for localization. Ears themselves have directional characteristics and processing of directional information takes places at an early stage of auditory networks in the central nervous system [1]. Typically, the direction of sound incidence is determined by neuronal computing of information from bilaterally arranged ears [1,2]. Two parameters are especially important for sound source localization: (i) interaural time differences (ITD) and (ii) interaural intensity differences (IID) [3,4]. Both parameters contribute to localization as, for example, humans use ITD for sounds of low pitch and IID for sounds of high pitch in the horizontal plane [1]. Some animals like owls even use the two different parameters for detection of different spatial components in sound source localization: IID for the vertical component or elevation and ITD for the horizontal component or azimuth [3]. This mechanism is made possible in owls by asymmetric ears. Other species have more symmetrical hearing systems, and also face the task for detection of the elevation of a sound source. In the median plane, an elevated sound source will not create temporal or intensity differences between the two ears in such hearing systems, however, spectral cues may be used for localization. For several mammalian hearing systems frequency-dependent transfer functions have been described, which create sound source position-dependent frequency differences at the tympanum [5–7]. Such transfer functions depend on the morphology of the system, e.g. of the head and the pinnae.

Sound source localization is a main task in acoustically communicating insects. The threshold sound pressure levels of afferents and interneurons typically are higher for stimuli from contralateral than for stimuli from ipsilateral [8,9]. However, in most insects there is no evidence that the nervous system can use the minute ITDs as directional cues [10]. Ears in these insects function primarily as pressure difference receivers, and multiple sound entrances create differences which can be used for directionality in the horizontal plane [4,8,10]. For detection of elevated sound source positions the mechanisms are less clear, but for example, it has been shown that phonotactically active parasitoid flies locate the sound source in three dimensions [11–14]. Biophysical and behavioural mechanisms might contribute to this location ability. Physiological and behavioural experiments have shown that bushcrickets can detect the position of calling conspecifics in three-dimensional habitats [15–18]. Bushcrickets have a sophisticated hearing system and might serve as an insect model system for hearing research [19,20]. The bushcricket ear has tonotopically arranged sensory cells within the proximal foreleg tibia [21–25]. The sensory units form a longitudinal row along a trachea which is connected to the tympana. The axons of the sensory cells run through the leg to the prothoracic ganglion where they connect to interneurons [26]. The peripheral hearing system is further characterized by distinct morphological structures. The sound waves enter the ‘acoustic spiracle’ at the thorax and are guided via an acoustic bulla and acoustic trachea to the tympanal organ in the foreleg [27,28]. Sound waves can also act directly on the tympanal membranes, which occur on the anterior and posterior side of the tibia [29–31]. Both, the spiracle and the tympanal membranes come in a variety of morphologies in the different species. For example, the spiracle can be occluded by the pronotum or it can be openly visible, whereby the spiracle opening correlates with hearing sensitivity [32,33]. Cuticular folds can cover the tympanal membranes and might contribute to directional hearing [27,34,35]. Defined by these external structures the auditory afferents in bushcrickets have directional response properties [34]. Here we tested the auditory tuning in respect to a three-dimensional hearing paradigm. A loudspeaker was moved in a spherical segment around the animal resulting in different test angles in azimuth and elevation, respectively. Threshold data were used to map the auditory space and to investigate whether such maps are species specific. The data were compared from three species of bushcrickets which have different morphologies of their hearing systems.

## 2. Material and methods

### 2.1 Animals and morphology

Three different bushcricket species were tested: (i) *Mecopoda elongata*, (ii) *Stilpnochlora couloniana* and (iii) *Ancylecha fenestrata*. All species were reared to the adult stages in large cages with fresh leaves (*A. fenestrata*, *S. couloniana*), seedlings and fish food (*M. elongata*). Animals were kept in a humid environment at 25°–28°C at a 12 L:12 D cycle. *M. elongata* (Mecopodinae) was bred in the laboratory for several generations, whereas *A. fenetrata* and *S. couloniana* (Phaneropterinae) were reared in first or second generation from commercially obtained nymphs.

For morphological investigations, the length of the tympana (proximo-distal direction) and the maximum opening width of the acoustic spiracle were determined. The structures were photographed with a Leica camera (2048×1028 px) attached to a dissecting microscope. Measuring was done with the software IMAGEJ.

Neural tracing of the tympanal nerve revealed the number of sensory cells in the crista acustica (for details see [36]). Basically, animals were fixed on a platform and the tympanal nerve was dissected in the femur. The nerve was cut and the distal end was placed in a glass capillary filled with 5% cobaltous chloride (aqueous solution). After 48 h, the leg was cut and the cobalt was precipitated with ammonium sulfide to reveal the marked structures [36]. Subsequently, the leg was dehydrated and placed in methylsalicylate for anatomical inspection. Preparations were photographed with a Leica camera (2048×1028 px) attached to a microscope (Olympus BH2).

### 2.2 Physiology

**Figure 1.**
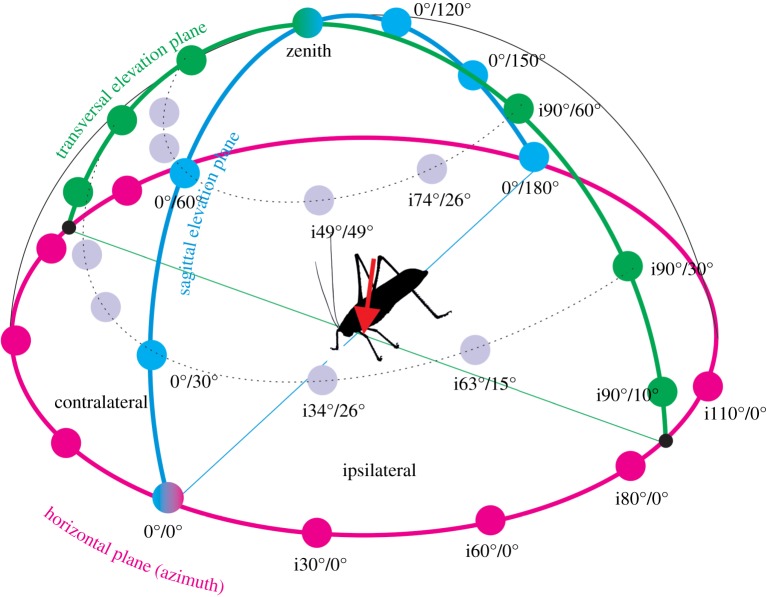
Schematic of the experimental set-up for stimulation and recording. The animal was placed in the centre of a circle with 25 cm distance to the loudspeaker. The positions of the loudspeaker in the azimuth are indicated in green letters (ipsilateral angles are indicated with ‘i’, contralateral with ‘c’). The loudspeaker positions in the vertical plane are indicated in blue letters. The recording site from the tympanal nerve in the left femur of the prothoracic leg is indicated with the red arrow. Drawing not to scale.

Adult animals were mounted upright on a narrow (3 mm width) metal holder with wax. The animals had a body width of 6.9–8.2 mm (at the thorax) and thereby concealed the metal holder. The femur of the foreleg was fixed 90° from the longitudinal axis of the body with a small wire (1 mm diameter) and the tibia with the tympanal organ was free in the sound field. The distance between tibia (ear) and the median body plane was 14–18 mm. Regarding the direction of the sound stimulation, the longitudinal body axis of the animal was orientated to 0° azimuth and 0° elevation (figure 1). The holder with the animal was mounted on a metal rod approximately 10 cm above echo minimizing foam (absorption coefficient greater than 0.98 for frequencies of 1 kHz and above) around the set-up. The recording electrodes (silver wire, 0.125 mm diameter; approx. 5 cm long) were placed in the left femur of the animals. The holder for the silver wires was 17 cm long and 7 mm diameter and attached to a holder approximately 14 cm distance behind the animal.

For the sound stimulation, a loudspeaker (Dynaudio DF21) was mounted on a half of a modified bicycle rim 25 cm distance to the animal. By tilting the rim, different angular positions around the centre where the animal was placed could be obtained. Sound source positions in three main planes in respect to the body orientation were analysed: in the horizontal plane (azimuth), the loudspeaker could be moved to archive test angles of i110°, i80°, i60° and i30° on the ipsilateral side, c30°, c60° and c80° on the contralateral side as well as the frontal position (0°; figure 1). In the vertical or median plane, test angles ranged from 0° to 180° covering a half circle in the sagittal plane (= at 0° azimuth) of the animal in 30° steps. In the vertical transversal plane (= at 90° azimuth), test angles ranged from 10° ipsilateral to 10° contralateral across the animal. Additional test angles were adjusted by raising the loudspeaker at ‘non-plane’ positions (from 30° and 60° azimuth) and completed the measurements of hearing space (figure 1). The whole set-up was placed in a Faraday cage covered with echo minimizing foam.

For recording, a small cut was made in the dorsal femur of the left foreleg. Care was taken not to damage any trachea and the tympanal nerve was placed on a hook electrode. A second electrode was inserted close to the recording electrode in the haemolymph of the femur. The nerve was kept moist with application of small droplets of locust ringer [37] and sealed with petroleum jelly. The recorded potentials were amplified 1000× (ISO-80 amplifier, WP Instruments) connected via an AD board (Micro-14013; Cambridge Electronic Design) to a computer for data storage. Data were stored and analysed with Spike2 (Cambridge Electronic Design).

Acoustic stimuli comprising pure sine waves between 4 and 40 kHz were generated with the Spike2 software. Each stimulus had a duration of 50 ms (1 ms rise/fall time) with a pause of 200 ms and was repeated five times. After a time delay of 400 ms, stimuli with the next intensity were presented. In pilot tests, the approximate thresholds for ipsilateral stimuli had been determined. For the experiments stimuli 10–15 dB sound pressure level (SPL) below and up to 20 dB SPL above the expected threshold were tested with 5 dB increments. Calibration of the SPL (rel. 20 μPa) was done on a continuous sound with a calibrated XL2 sound analyser (NTI Audio Instruments) and 1/4 inch calibrated microphones (M2210, NTI Audio Instruments and SR40, Earthworks, connected via Tascam US-600 to a computer).

For data analyses, thresholds were determined visually as the sound intensity at which neuronal activity was seen in response to at least four of the five stimulus repetitions. For averaging threshold, SPL data were transferred to sound pressure (rel. 20 μPa) and thereafter transformed back to dB SPL for visualization. The mean threshold data were also depicted in contour graphs, whereby the threshold is colour coded. The contour was created from (*x*,*y*,*z*) coordinates (frequency, position, threshold) and interpolated with Origin 9.0 (Originlab).

In order to visualize threshold changes in auditory space, all measured threshold values were related to the hearing threshold in the frontal position (0° azimuth; 0° elevation). Reduction in threshold or increase in threshold was colour coded for each frequency and depicted in a space diagram for each species.

For the calculations of the suprathreshold response strength, the modulus of the recording trace was calculated in Spike2. The sound-evoked activity 60 ms after stimulus onset was compared with the background activity of the nerve determined in a 60 ms window before the stimulus (see the electronic supplementary material, S1 for illustration). The resulting values were plotted as percentage of the background activity for different stimulus angles.

## 3. Results

### 3.1 Morphology

**Figure 2.**
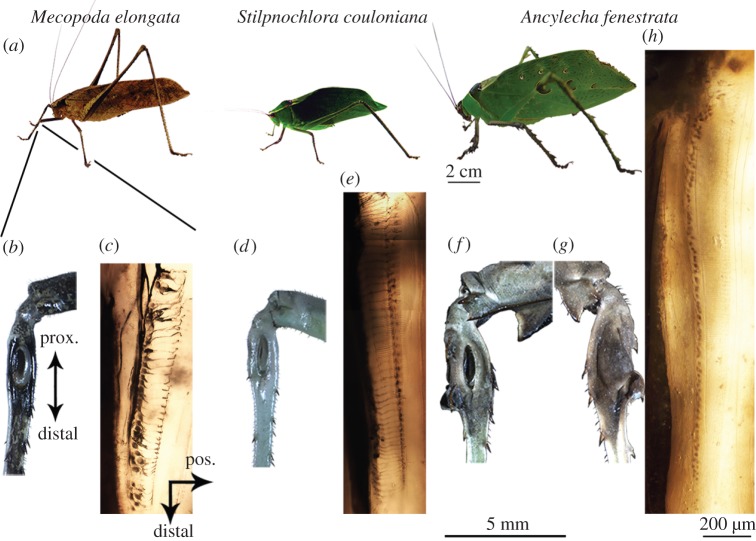
Experimental animals and their peripheral hearing systems. (*a*–*c*) *Mecopoda elongata*. The position of the ear is indicated by the lines to the left front leg. The tympanum is open (= not covered by cuticular flaps; *b*, posterior view). Neuronal tracing reveals the row of scolopidial receptor cells and their dendrites in the crista acustica (*c*). (*d*,*e*) *Stilpnochlora couloniana*. The tympanum is open (*d*) and the crista acustica comprises more sensory cells (see the electronic supplementary material, S2) than *M. elongata* (*e*). (*f*–*h*) *Ancylecha fenestrata*. Whereas the posterior tympanum is open (*f*), the anterior tympanum is occluded by a cuticular flap (*g*). The crista acustica contains a large number (see the electronic supplementary material, S2) of scolopidial units (*h*, only sensory cell bodies are labelled). Scales for all three parts of the figure are given at *A. fenestrata*, for the animal size (2 cm), the tympanum size (5 mm) and the ear size (200 μm).

The auditory systems of the investigated species exhibit species-specific morphological characters (figure 2). The opening of the acoustic spiracle is freely visible in *M. elongata*, but occluded by the pronotum in *S. couloniana* and *A. fenestrata*. *Mecopoda elongata* and *S. couloniana* have open tympana, whereas in *A. fenestrata* a pronounced cuticular flap covers the anterior tympanum. All three species possess a typical arrangement of auditory receptor cells, with *A. fenestrata* exhibiting by far the highest number of sensory units (see the electronic supplementary material, S2 for morphometric data).

### 3.2 Comparison of species-specific hearing thresholds

**Figure 3.**
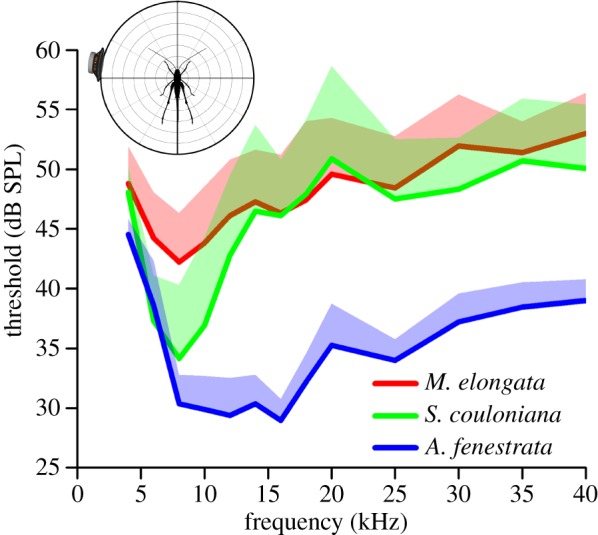
Average hearing thresholds of the three species in respect to horizontal ipsilateral stimulation (i80°). The three species have clearly distinct threshold curves. Given are the mean thresholds (bold lines; *n*=10–11 for *M. elongata* and *S. couloniana*, *n*=8 for *A. fenestrata*; data of males and females), with one standard deviation depicted to higher SPL values.

Extracellular recordings of the tympanal nerve activity during pure-tone stimulation from a stimulus direction of i80° (80° ipsilateral and 0° vertical; 80° instead of 90° was used because of mechanical constraints of the set-up) show species-specific hearing thresholds (figure 3). The threshold of *M. elongata* is rather flat from 4 kHz up to 40 kHz with a minimum at approximately 8 kHz. The threshold curve of *S. couloniana* has a pronounced minimum at 8 kHz, but is about 7 dB more sensitive with a threshold of about 35 dB SPL. The most-sensitive hearing was recorded from *A. fenestrata* with a broad minimum range from 8 kHz up to 16 kHz with a threshold of approximately 30 dB SPL. The thresholds of males and females were similar, except for a few frequencies in two of five loudspeaker positions in *S. couloniana* (see the electronic supplementary material, S3 for *p*-values).

### 3.3 Hearing thresholds of the three different species in relation to different loudspeaker positions

**Figure 4.**
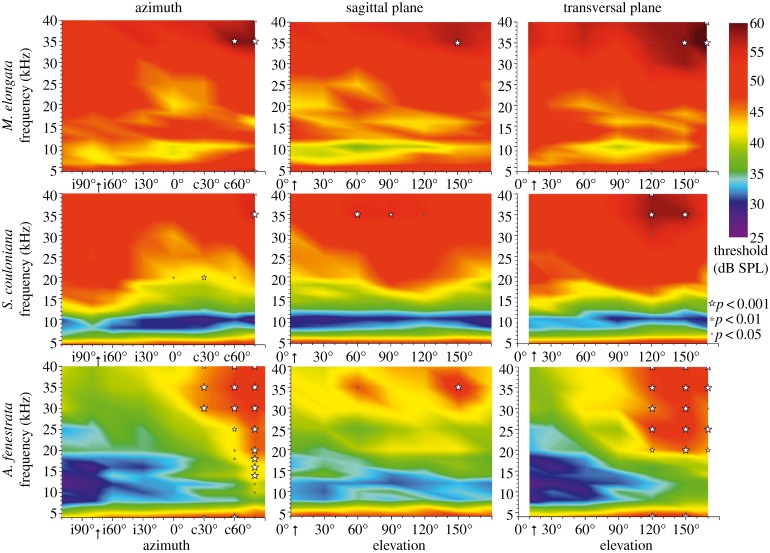
Colour-coded thresholds values (in dB SPL) for carrier frequencies from 4 to 40 kHz along three different planes in space for all three tested species. Low threshold values are bluish-purple, high threshold values are red (see scheme at top right). The data points were plotted in a matrix and intermediate values are calculated with the software Origin Pro9.0. Each data point represents the mean from 8 to 14 measurements. Statistically significant differences from positions in space compared with the threshold values at i80° (0° elevation) for azimuth, 0° (0° azimuth) for sagittal elevation and 10° (i90° azimuth) for transversal elevation are indicated by a ‘star’. The size of the ‘star’ indicates the *p*-value (two-way ANOVA, Bonferroni post test; scheme at right margin). The data show that the three species have a distinct pattern of thresholds within the different planes. The threshold values depend on the position in space.

**Figure 5.**
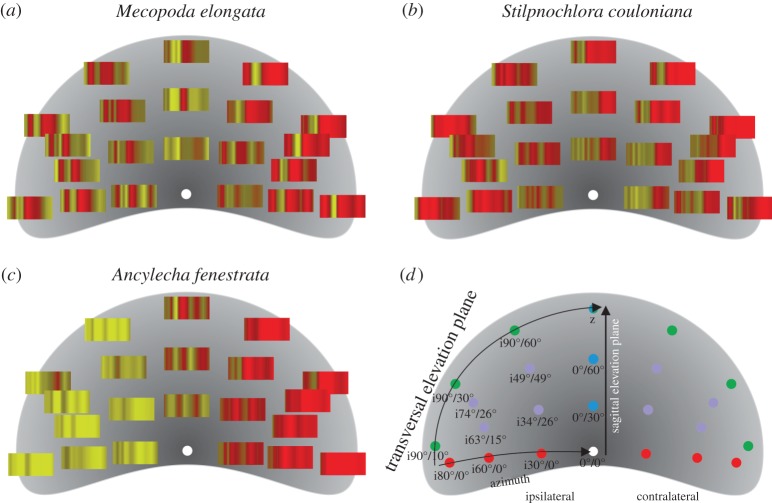
Colour-coded *changes* in thresholds in the auditory space for the three species (*a*–*c*). The tested loudspeaker positions in the fronto-dorsal auditory space of the animal are indicated in (*d*). The changes in threshold have been calculated in respect to the threshold at the frontal position (0°,0°) with yellow coding for lower threshold values, red for higher values; focus is on relative differences, therefore no scaling is presented; the *x*-axis of each rectangle extends from 4 to 40 kHz. The figure visualizes that each species has a distinct auditory space based on its relative threshold changes, which might indicate the position of a sound source to the animal.

The hearing sensitivity in all three tested species depends to different degree on the position of the sound source. In order to visualize this, the hearing thresholds were colour coded and plotted for all frequency-position combinations in the three main planes in space (figure 4): it becomes obvious that each species has a specific hearing threshold in space. *Mecopoda elongata* has relatively high thresholds for all loudspeaker positions (reflected by the red colour in figure 5). The thresholds are relatively uniform in space, whereby lowest values were recorded for frequencies around 10 kHz at a few positions (at approx. 60° vertical elevation). Significant differences within one plane are found only at a few positions (marked with stars; threshold values were compared with those from positions in the respective plane marked with an arrow in figure 4). *Stilpnochlora couloniana* has a narrow band of best hearing from 7–12 kHz in all tested positions (bluish areas in figure 5). Significant differences of threshold values within all three planes can be found at a few but different positions (figure 4). Largest differences of position specific hearing thresholds were found in *A. fenestrata*. In the horizontal and transversal plane, the best hearing range is clearly ipsilateral of the animal. Most of the significant differences are found in the high frequency range, above 20 kHz in all three planes.

Visualization of threshold changes in the complete fronto-dorsal auditory space of each species shows position-dependent hearing (figure 5). The threshold changes are calculated relative to the threshold for sounds from the frontal position (0° azimuth; 0° elevation). The colour coding indicates that relative lower thresholds (yellow) are mainly found on the ipsilateral side, whereas increases in thresholds (red) are on the contralateral side. However, not all frequencies and species follow this trend. In *S. couloniana* position-independent threshold changes and increases in threshold are mainly found in the high frequency range.

### 3.4 Position-dependent suprathreshold responses

**Figure 6.**
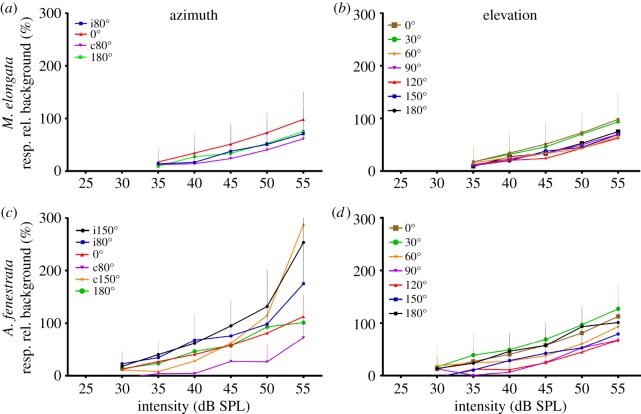
Suprathreshold intensity responses to sound stimuli with 20 kHz carrier frequency presented from different sound incidences. The exemplary suprathreshold responses are expressed as a percentage of activity above background nerve activity (further explanation in Material and methods and the electronic supplementary material, figure S1). Responses increase linearly (*a*,*b*,*d*) and nonlinearly (*c*) with increasing sound intensity. *n*=3–9 (*A. fenestrata*), *n*=7–12 (*M. elongata*).

Since hearing properties are not reflected only in the threshold, suprathreshold responses of the sound-induced tympanal nerve activity have been calculated (see Material and methods for details). The response to stimuli is plotted as a percentage above the baseline activity. Suprathreshold responses at a stimulus frequency of 20 kHz up to 25 dB above threshold were depicted for two species (figure 6). Data of *M. elongata* show, independent of the frequency and loudspeaker position, a linear increase in the neuronal response with different slopes (figure 6*a*,*b*). The response of *S. couloniana* was similar (data not shown). However, for *A. fenestrata*, a nonlinear increase with azimuthal position-dependent factors is seen at higher stimulus intensities (figure 6*d*), whereas for vertical positions only a linear increase is found (figure 6*c*).

## 4. Discussion

We investigated peripheral hearing thresholds of three species of bushcrickets in respect to different loudspeaker positions. Firstly, the results show species specific hearing thresholds. Species-specific hearing thresholds have been reported for a number of tettigonid species. Typically, thresholds correlate roughly with the peak frequency of the intraspecific calling songs [33,38–40]. The three investigated species have different broadbanded spectra in their male calling songs in the frequency range up to 96 kHz (see the electronic supplementary material, S4 for spectra). *Mecopoda elongata* has a rather broad spectrum from 7 to 70 kHz correlating to the broad hearing threshold, although for *M. elongata* also a tuning of the hearing threshold to frequencies of 15–16 kHz has been reported [31,41]. The hearing ranges of *S. couloniana* and *A. fenestrata* also match to the peak frequencies of the spectrum of the respective calling songs. Nevertheless, such matches have to be considered cautiously, as the behaviourally important frequency domains are not known. Filtering in the habitat might influence the signal and additional processes like parasite pressure might influence the hearing threshold [40,42].

Secondly, the hearing thresholds of all three tested species depend on the sound source location. As early as 1940, Autrum showed that the threshold for ipsilateral sound sources is lower than for contralateral sound sources [8]. Since then, directional responses were noted on different neuronal levels from peripheral response, to interneurons and behaviour in Ensifera and other insects [18,43,44]. Biophysical investigations showed that the directionality for crickets is best at frequencies close to the peak frequency of the calling song [4]. The peripheral directionality is translated to interneurons, whereby presynaptic inhibition and reciprocal inhibitory connections reinforce directional responses [26,45,46]. This neuronal activity is the basis for directional behavioural responses [18,44,47]. Here we have extended physiological experiments to elevated sound source positions and show that the threshold curves depend on the frequency in combination with the sound source position in a graded fashion. These position effects on the hearing threshold are mainly found in the high frequency range and are species specific.

Localization of elevated sound sources in vertebrates can be explained by transfer functions of sound waves along structures like the pinnae which guide and filter sound waves [1]. The resulting transfer functions (head-related transfer functions) are often responsible for locating elevated sound sources as the pinnae transfer the frequencies differently depending on the sound incidence to the tympanal membrane [1,5,7]. In other animals, like alligators which lack elaborated morphologies influencing sound waves, generation of pressure differences are responsible for localization [48].

Tettigoniids have distinct morphologies of the auditory system. Our results indicate that morphology is important, as the three species have distinct auditory spaces. Typically, tettigoniids have four sound entrances [49]: the ipsilateral and contralateral acoustic spiracles, and the tympana on both forelegs. The acoustic trachea is the principle input of acoustic energy with distinct species-specific transfer functions and functioning like an exponential horn [50]. Similarly, the spiracle morphology is not uniform in tettigoniids, with species that have openly visible spiracles, like *M. elongata*, whereas in others the pronotum covers the opening, like in *S. couloniana* and *A. fenestrata*. Other species can even actively close the spiracle [51].

Furthermore, the tympanal organ in the tibia is differently structured. The tympana can be openly exposed, as in the Phaneropteridae or covered by cuticular flaps [27]. The cuticular cover can be formed such that only small slits are open or cuticular flaps may be present only on one side, like in *A. fenestrata*. The slits and the spiracles are responsible for coding directionality [34,52]. Modifications, like dampening of the sound transmission in the acoustic trachea and occlusion of slits result in loss of directionality in distinct frequency bands [52]. Behavioural tests showed that slits function in directed phonotaxis, whereby these tests have only been performed in a flat two-dimensional arena [35].

All these peripheral structures, including the animal's body shape, might influence hearing and coding of auditory space. We have investigated the tuning of the afferents of one ear in the three species, whereby care has been taken to disturb the hearing system as little as possible. The animal holder including the recording electrode was relatively small and differences in thresholds are likely to be caused by attenuation and diffraction at the animal's body.

Here we showed that in the auditory space the peripheral tuning is affected by the position. Thus, the thresholds of some frequencies are changed, whereas others are relatively unaffected. The tympanal membranes vibrate in complex modes in relation to sound input [29,53] and peripheral receptor cells detect these vibrations. The receptor cells form a linear row with a peripheral tonotopy [23,25,54,55]. Among insects, tettigoniids have perhaps the most elaborated frequency fractioning in the ear, although the frequency tuning gets somewhat lost in the central nervous system, where the 30–80 receptor cells converge on a low number of auditory interneurons [26,56]. The tuning and directionality of the interneurons is based on integration of excitatory and inhibitory connections [57]. Therefore, the neuronal responses of the afferents, including their presynaptic inhibition [45,58] will influence the responses of the interneurons despite the overall convergence of frequency information and will influence the localization behaviour of the animal. Processing of information from both ears will even strengthen the possibility of locating a sound source in space.

The suprathreshold responses of the afferents typically showed a linear increase for all frequencies, carrying forward the position-dependent neuronal excitation into the central nervous system. Some responses (in *A. fenestrata*) even had a nonlinear increase above threshold which might amplify differences between frequencies depending on the sound source position. In the suprathreshold range also additional factors like intensity fractioning of afferents [46] or non-monotonic response properties [58] will affect directional hearing. Such properties are important for auditory information processing and influence hearing physiology [40].

All these differences might provide cues for the animals for the sound source location in a three-dimensional space. It has been shown, that tettigoniids, can behaviourally locate a sound source in three dimensions [15,16]. Physiologically, raising a loudspeaker above the horizon resulted in different neuronal responses (see also [18,34]). On the other hand, in their habitat the situation is more complex, with animals standing on a branch or leaf of a tree, and perceiving sounds which are reflected and modified through the habitat [42]. Furthermore, during walking the leg position will constantly change and might influence hearing even further [59].

The three species seem to have position-dependent differences in hearing thresholds, correlating to the different morphologies. *Ancylecha fenestrata* with a covered anterior tympanum shows the largest differences. Although we did not manipulate the hearing system, the different responses indicate the importance of these structures. In some species, morphological asymmetries have been detected in the hearing system, for example, in the spiracle size [38,60]. For phonotaxis in flat arenas this asymmetry was found to be not very important [60]. However, such asymmetries could be important for hearing in three-dimensional space, in analogy to the asymmetric hearing system of the barn owl [3]. For the tettigoniid, it might not result in a separation of ITD and IID, but in a side-specific tuning which might correlate to the sound-source position.

Speculatively, the position-dependent frequency tuning of hearing might also be a driving force for the frequency fractioning and tonotopy in the tettigoniid hearing organ. While many insects have the possibility of frequency discrimination [61], the tettigoniids have the above described linear row of sensory cells and the corresponding frequency fractioning in the ear. The evolution of this character might be related to the necessary detection of sound source localizations in a complex three-dimensional habitat. Tettigoniids often live in bushes and trees and therefore have the task of finding mates in three-dimensional space. Nevertheless, more ecological and behavioural data are necessary to understand auditory three-dimensional localization.

In conclusion, species-specific tuning depending on frequency and position has been found in the peripheral hearing threshold. This study opens possibilities of analysing the contribution of the morphological characters for frequency-dependent diffraction of sound waves or to correlate physiology with behaviour.

## Supplementary Material

1 Method: Calculation of the suprathreshold response strength

## Supplementary Material

2 Table with morphometric data

## Supplementary Material

3 Table with P values of hearing thresholds between males and females

## Supplementary Material

4 Figure with characters of the calling songs of the experimental species

## Data accessibility

Data used for the calculations of the physiological results are available from Dryad, http://dx.doi.org/10.5061/dryad.kj731.

## References

[1] Blauert J 1996 Spatial hearing. Cambridge, UK: MIT Press.

[2] Popper A, Fay R 2005 Sound source localization. Berlin, Germany: Springer.

[3] Konishi M 1993 Neuroethology of sound localization in the owl. J. Comp. Physiol. A 173, 3–7. (doi:10.1007/BF00209613)

[4] Michelsen A, Popov AV, Lewis B 1994 Physics of directional hearing in the cricket *Gryllus bimaculatus*. J. Comp. Physiol. A 175, 153–164. (doi:10.1007/BF00215111)

[5] Rice J, May B, Spirou G, Young E 1992 Pinna-based spectral cues for sound localization in cat. Hearing Res. 58, 132–152. (doi:10.1016/0378-5955(92)90123-5) 10.1016/0378-5955(92)90123-5 1568936

[6] Chen Q, Cain D, Jen P 1995 Sound pressure transformation at the pinna of *Mus domesticus*. J. Exp. Biol. 198, 2007–2023. 7595162 10.1242/jeb.198.9.2007

[7] Lauer A, Slee S, May B 2011 Acoustic basis of directional acuity in laboratory mice. J. Ass. Res. Otolaryng. 12, 633–645. (doi:10.1007/s10162-011-0279-y) 10.1007/s10162-011-0279-y PMC3173556 21717290

[8] Autrum H 1940 Über Lautäusserungen und Schallwahrnehmung bei Arthropoden II. Das Richtungshören von Locusta und Versuch einer Hörtheorie für Tympanalorgane vom Locustidentyp. Z. Vergl. Zool. 20, 326–352. (doi:10.1007/BF00342439)

[9] Kalmring K, Rheinlaender J, Römer H 1972 Akustische Neurone im Bauchmark von *Locusta migratoria*—Der Einfluß der Schallrichtung auf die Antwortmuster. J. Comp. Physiol. 80, 325–352. (doi:10.1007/BF00694845)

[10] Michelsen A 1994 Directional hearing in crickets and other small animals. In Neural basis of behavioural adaptations (eds Schildberger K, Elsner N), pp. 195–207. Stuttgart, Germany: Gustav Fischer.

[11] Ramsauer N, Robert D 2000 Free-flight phonotaxis in a parasitoid fly: behavioural thresholds, relative attraction and susceptibility to noise. Naturwissenschaften 87, 315–319. (doi:10.1007/s001140050729) 11013880 10.1007/s001140050729

[12] Müller P, Robert D 2001 A shot in the dark: the silent quest of a free-flying phonotactic fly. J. Exp. Biol. 204, 1039–1052. 11222123 10.1242/jeb.204.6.1039

[13] Lakes-Harlan R, Köhler U 2003 Influence of habitat structure on phonotactic strategy of a parasitoid fly (*Emblemasoma auditrix*, Sarcophagidae, Diptera). Ecol. Entomol. 28, 758–765. (doi:10.1111/j.1365-2311.2003.00562.x)

[14] Arthur B, Hoy R 2006 The ability of the parasitoid fly *Ormia ochracea* to distinguish sounds in the vertical plane. J. Acoust. Soc. Am. 120, 1546–1549. (doi:10.1121/1.2225936) 17004476 10.1121/1.2225936

[15] Ofner E, Rheinlaender J, Römer H 2007 Spatial orientation in the bushcricket *Leptophyes punctatissima* (Phaneropterinae; Orthoptera). II. Phonotaxis to elevated sound sources on a walking compensator. J. Comp. Physiol. A 193, 321–330. (doi:10.1007/s00359-007-0210-5) 10.1007/s00359-007-0210-5 17273848

[16] Rheinlaender J, Hartbauer M, Römer H 2007 Spatial orientation in the bushcricket *Leptophyes punctatissima* (Phaneropterinae; Orthoptera). I. Phonotaxis to elevated and depressed sound sources. J. Comp. Physiol. A 193, 313–320. (doi:10.1007/s00359-006-0186-6) 10.1007/s00359-006-0186-6 17086427

[17] Rheinlaender J, Shen J-X, Römer H 2006 Auditory lateralization in bushcrickets: a new dichotic paradigm. J. Comp. Physiol. A 192, 389–397. (doi:10.1007/s00359-005-0078-1) 10.1007/s00359-005-0078-1 16362304

[18] Kostarakos K, Rheinlaender J, Römer H 2007 Spatial orientation in the bushcricket *Leptophyes punctatissima* (Phaneropterinae; Orthoptera). III. Peripheral directionality and central nervous processing of spatial cues. J. Comp. Physiol. A 193, 1115–1123. (doi:10.1007/s00359-007-0262-6) 10.1007/s00359-007-0262-6 17713767

[19] Montealegre-Z F, Jonsson T, Robson-Brown K, Postles M, Robert D 2012 Convergent evolution between insect and mammalian audition. Science 338, 968–971. (doi:10.1126/science.1225271) 23162003 10.1126/science.1225271

[20] Udayashankar A, Kössl M, Nowotny M 2012 Tonotopically arranged traveling waves in the miniature hearing organ of bushcrickets. PLoS ONE 7, 31008 (doi:10.1371/journal.pone.0031008) 10.1371/journal.pone.0031008 PMC3278424 22348035

[21] Schwabe J 1906 Beiträge zur Morphologie und Histologie der tympanalen Sinnesapparate der Orthopteren. Zoologica 50, 1–154.

[22] Lakes R, Schikorski T 1990 Neuroanatomy of Tettigoniids. In The Tettigoniidae: biology, systematics and evolution (eds Bailey W, Rentz D), pp. 166–190. Bathurst, Australia: Crawford House Press.

[23] Oldfield B 1982 Tonotopic organisation of auditory receptors in Tettigoniidae (Orthoptera: Ensifera). J. Comp. Physiol. 147, 461–469. (doi:10.1007/BF00612011)

[24] Römer H 1983 Tonotopic organization of the auditory neuropile in the bushcricket *Tettigonia viridissima*. Nature 306, 60–62. (doi:10.1038/306060a0)

[25] Stumpner A 1996 Tonotopic organization of the hearing organ in an bushcricket. Naturwis- senschaften 83, 81–84. (doi:10.1007/bf01141875)

[26] Römer H, Marquart V, Hardt M 1988 Organization of a sensory neuropile in the auditory pathway of two groups of orthopterans. J. Comp. Neurol. 275, 201–215. (doi:10.1002/cne.902750204) 3220974 10.1002/cne.902750204

[27] Bailey W 1990 The ear of the bushcricket. In The Tettigoniidae. biology, systematics and evolution (eds Bailey W and Rentz DCF), 217–247. Bathurst, Australia: Crawford House Press.

[28] Michelsen A, Heller K-G, Stumpner A, Rohrseitz K 1994 The gain of the auditory trachea in bushcrickets, determined with a new method. J. Comp. Physiol. A 175, 145–151. (doi:10.1007/BF00215110) 8071893 10.1007/BF00215110

[29] Michelsen A, Larsen O 1978 Biophysics of the ensiferan ear. I. Tympanal vibrations in the bushcrickets (Tettigoniidae) studies with laser vibrometry. J. Comp. Physiol. A 123, 193–203. (doi:10.1007/BF00656872)

[30] Bangert M, Kalmring K, Sickmann T, Stephen R, Jatho M, Lakes-Harlan R 1998 Stimulus transmission in the auditory receptor organs of the foreleg of bushcrickets (Tettigoniidae). I. The role of the tympana. Hearing Res. 115, 27–38. (doi:10.1016/S0378-5955(97)00177-9) 10.1016/s0378-5955(97)00177-9 9472733

[31] Hummel J, Kössl M, Nowotny M 2011 Sound-induced tympanal membrane motion in bushcrickets and its relationship to sensory output. J. Exp. Biol. 214, 3596–3604. (doi:10.1242/jeb.054445) 21993788 10.1242/jeb.054445

[32] Stumpner A, Heller K-G 1992 Morphological and physiological differences of the auditory system in three related bushcrickets (Orthoptera: Phaneropteridae, *Poecilimon*). Physiol. Entomol. 17, 73–80. (doi:10.1111/j.1365-3032.1992.tb00992.x)

[33] Dobler S, Stumpner A, Heller K-G 1994 Sex-specific spectral tuning for the partner's song in the duetting bushcricket *Ancistrura nigrovittata* (Orthoptera: Phaneropteridae). J. Comp. Physiol. A 175, 303–310. (doi:10.1007/BF00192989)

[34] Bailey W, Stephen R 1978 Directionality and auditory slit function: a theory of hearing in bushcrickets. Science 201, 633–634. (doi:10.1126/science.201.4356.633) 17794126 10.1126/science.201.4356.633

[35] Bailey W, Stephen R 1984 Auditory acuity in the orientation behaviour of the bushcricket *Pachysagella australis* Walker (Orthoptera, Tettigoniidae, Saginae). Anim. Behav. 32, 816–829. (doi:10.1016/S0003-3472(84)80158-X)

[36] Strauß J, Lehmann G, Lehmann A, Lakes-Harlan R 2012 Spatial organization of tettigoniid auditory receptors: insights from neuronal tracing. J. Morph. 273, 1280–1290. (doi:10.1002/jmor.20058) 22807283 10.1002/jmor.20058

[37] Clements A, May T 1974 Studies on locust neuromuscular physiology in relation to glutamic acid. J. Exp. Biol. 60, 673–705. 4367892 10.1242/jeb.60.3.673

[38] Bailey W, Römer H 1991 Sexual differences in auditory sensitivity: mismatch of hearing threshold and call frequency in a tettigoniid (Orthoptera, Tettigoniidae: Zaprochilinae). J. Comp. Physiol. A 169, 349–353. (doi:10.1007/BF00206999)

[39] Riede K, Kämper G, Höfler I 1990 Tympana, auditory thresholds, and projection areas of tympnala nerves in singing and silent grasshoppers (Insecta, Acridoidea). Zoomorphology 109, 223–230. (doi:10.1007/BF00312473)

[40] Schul J, Patterson A 2003 What determines the tuning of hearing organs and the frequency of calls? A comparative study in the katydid genus *Neoconocephalus* (Orthoptera, Tettigoniidae). J. Exp. Biol. 206, 141–152. (doi:10.1242/jeb.00070) 12456704 10.1242/jeb.00070

[41] Hartbauer M, Radspieler G, Römer H 2010 Reliable detection of predator cues in afferent spike trains of a katydid under high background noise levels. J. Exp. Biol. 213, 3036–3046. (doi:10.1242/jeb.042432) 20709932 10.1242/jeb.042432 PMC3971152

[42] Römer H 1993 Environmental and biological constraints for the evolution of long-range signalling and hearing in acoustic insects. Phil. Trans. R. Soc. Lond. B 226, 179–185. (doi:10.1098/rstb.1993.0056)

[43] Hill K, Boyan G 1977 Sensitivity to frequency and direction of sound in the auditory system of crickets (Gryllidae). J. Comp. Physiol. 121, 79–97. (doi:10.1007/BF00614182)

[44] von Helversen D 1997 Acoustic communication and orientation in grasshoppers. In Orientation and communication in arthropods (ed. Lehrer M), pp. 301–343. Basel, Germany: Birkhäuser.

[45] Baden T, Hedwig B 2010 Primary afferent depolarization and frequency processing on auditory afferents. J. Neurosci. 30, 14 862–14 869. (doi:10.1523/JNEUROSCI.2734-10.2010) 10.1523/JNEUROSCI.2734-10.2010 PMC6633615 21048145

[46] Hennig RM, Franz A, Stumpner A 2004 Processing of auditory information in insects. Microsc. Res. Tech. 63, 351–374. (doi:10.1002/jemt.20052) 15252878 10.1002/jemt.20052

[47] Pollack GS 2000 Who, what, where? Recognition and localization of acoustic signals by insects. Curr. Opin. Neurobiol. 10, 763–767. (doi:10.1016/S0959-4388(00)00161-6) 11240287 10.1016/s0959-4388(00)00161-6

[48] Bierman H, Thornton J, Jones H, Koka K, Young B, Brandt C, Christensen-Dalsgaard J, Carr C, Tollin D 2014 Biophysics of directional hearing in the American alligator (*Alligator mississippiensis*). J. Exp. Biol. 217, 1094–1107. (doi:10.1242/jeb.092866) 24671963 10.1242/jeb.092866 PMC3966920

[49] Michelsen A 1998 Biophysics of sound localization in insects. In Comparative hearing: insects (eds Hoy RR, Popper AN, Fay RR), pp. 18–62. New York, NY: Springer.

[50] Rössler W, Jatho M, Kalmring K 2006 The auditory-vibratory sensory system in bushcrickets. In Insect sound and communication (eds Drosopoulos S, Claridge MF), pp. 35–69. Boca Raton, FL: CRC Press

[51] Römer H, Bailey W 1998 Strategies for hearing in noise: peripheral control over auditory sensitivity in the bushcricket *Sciarasaga quadrata* (Austrosaginae: Tettigoniidae). J. Exp. Biol. 201, 1023–1033. 9487106 10.1242/jeb.201.7.1023

[52] Mason AC, Morris G, Wall P 1991 High ultrasonic hearing and tympanal slit function in rainforest katydids. Naturwissenschaften 78, 365–367. (doi:10.1007/BF01131611)

[53] Nowotny M, Hummel J, Weber M, Möckel D, Kössl M 2010 Acoustic-induced motion of the bushcricket (*Mecopoda elongata*, Tettigoniidae) tympanum. J. Comp. Physiol. A 196, 939–945. (doi:10.1007/s00359-010-0577-6) 10.1007/s00359-010-0577-6 20827480

[54] Oldfield BP 1988 Tonotopic organization of the insect auditory pathway. TINS 11, 267–270. (doi:10.1016/0166-2236(88)90108-7) 2465624 10.1016/0166-2236(88)90108-7

[55] Stölting H, Stumpner A 1998 Tonotopic organization of auditory receptors of the bushcricket *Pholidoptera griseoptera* (Tettigoniidae, Decticinae). Cell Tissue Res. 294, 377–386. (doi:10.1007/s004410051187) 9799453 10.1007/s004410051187

[56] Stumpner A, von Helversen D 2001 Evolution and function of auditory system in insects. Naturwissenschaften 88, 159–170. (doi:10.1007/s001140100223) 11480703 10.1007/s001140100223

[57] Rheinlaender J, Römer H 1980 Bilateral coding of sound direction on the CNS of the bushcricket *Tettigonia viridissima* L. (Orthoptera, Tettigoniidae). J. Comp. Physiol. A 140, 101–111. (doi:10.1007/BF00606302)

[58] Sippel M, Breckow J 1984 Non-monotonic response-intensity characteristics of auditory receptor cells in *Locusta migratoria*. J. Comp. Physiol. A 155, 633–638. (doi:10.1007/BF00610849)

[59] Hedwig B 2006 Pulses, patterns and paths: neurobiology of acoustic behaviour in crickets. J. Comp. Physiol. A 192, 677–689. (doi:10.1007/s00359-006-0115-8) 10.1007/s00359-006-0115-8 16523340

[60] Bailey W, Yang S 2002 Hearing asymmetry and auditory acuity in the Australian bushcrickets *Requena verticalis* (Listroscelidinae; Tettigoniidae; Orthoptera). J. Exp. Biol. 205, 2935–2942. 12177158 10.1242/jeb.205.18.2935

[61] Strauß J, Lakes-Harlan R 2014 Evolutionary and phylogenetic origins of tympanal hearing organs in insects. In Insect hearing and acoustic communication (ed. Hedwig B), pp. 5–26. New York, NY: Springer.

