# Supplementary material for: Position-dependent hearing in three species of bushcrickets (Tettigoniidae, Orthoptera)
Source: R Soc Open Sci. 2015 Jun 9;2(6):140473. doi: 10.1098/rsos.140473 (PMC4632538; doi:10.1098/rsos.140473)
Supplement: 1 Method: Calculation of the suprathreshold response strength [file rsos140473supp1.docx]

**Supplementary Information 1**

Calculation of the suprathreshold response strength.

The extracellular recording was analysed with the software Spike2 (example below). The modulus of the recording trace (SAP: summated action potential) was calculated for 60ms each at two time points: First, starting 70 ms before a stimulus and secondly starting with stimulus onset. The recording before the stimulus described the background activity of the nerve. After a 10ms pause the response to the auditory stimulus is calculated and expressed as percentage of the background (baseline) activity.

Data of the example trace below: *Ancylecha fenestrata*, loudspeaker position: ipsilateral 80°, elevation 0°, stimulus duration: 50 ms, carrier frequency of the stimulus: 20 kHz, 75 dB SPL. The modulus of the response is 164 % of the background activity.

| 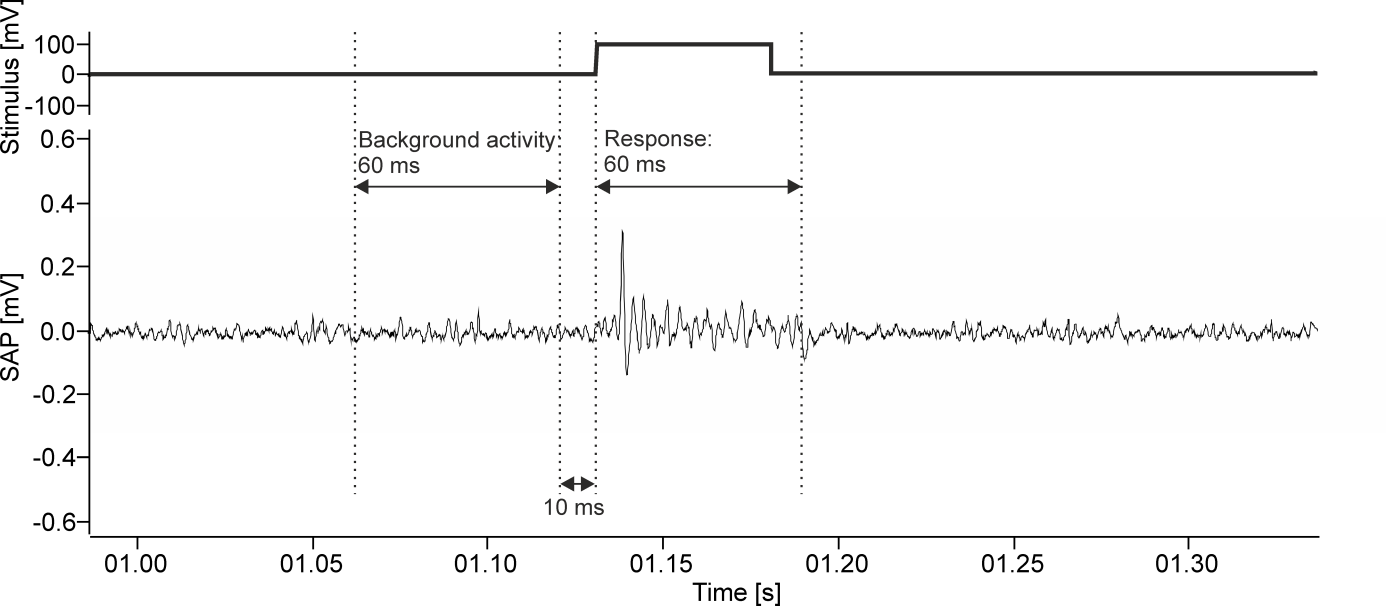 |
| --- |
