# Supplementary material for: Position-dependent hearing in three species of bushcrickets (Tettigoniidae, Orthoptera)
Source: R Soc Open Sci. 2015 Jun 9;2(6):140473. doi: 10.1098/rsos.140473 (PMC4632538; doi:10.1098/rsos.140473)
Supplement: 2 Table with morphometric data [file rsos140473supp2.docx]

|  | *M. elongata* | | *A. fenestrata* | | *S. couloniana* | |
| --- | --- | --- | --- | --- | --- | --- |
|  | ♂ | ♀ | ♂ | ♀ | ♂ | ♀ |
| Spiracle (mm) | free | | occluded | | occluded | |
|  | 2.7 (±0.24)  n = 5 | 3.0 (±0.32)  n = 8 | 3.7 (±0.48)  n = 5 | 3.7  (±0.48)  n = 3 | 4.6  (±0.35)  n = 5 | 4.2  (±0.21)  n = 5 |
| Tympanum  (anterior, mm) | open | | occluded | | open | |
|  | 1.74 (±0.14)  n = 5 | 1.86 (±0.13)  n = 7 | 2.4 (±0.15)  n = 8 | 2.09  (±0.18)  n = 6 | 1.82  (±0.03)  n = 4 | 1.56  (±0.1)  n = 7 |
| Tympanum  (posterior, mm) | open | | ventral bulge | | open | |
|  | 1.74 (±0.13)  n = 5 | 1.83 (±0.14)  n = 7 | 1.88 (±0.10)  n = 8 | 1.8  (±0.11)  n = 6 | 1.67  (±0.02)  n = 4 | 1.47  (±0.05)  n = 7 |
| Sensory cells (n) | 46-48* | | >80 | | 45-55 | |

**Supplementary Information 2**

Morphometric data of the auditory systems of the different species (mean with SD). For the spiracle the largest diameter was determined. The spiracle opening is with an average diameter of 4.2 mm largest in *S. couloniana* and with 2.1 mm smallest in *A. fenestrata*. Spiracles of *A. fenestrata* and *S. couloniana* are occluded by the pronotum. The tympana were measured in their proximo-distal orientation. The sizes of the tympanal membranes vary from 1.47 mm up to 1.86 mm in length, with *S. couloniana* having the smallest tympana. The anterior tympanum of *A. fenestrata* is occluded by a cuticular flap. The number of sensory cells was analysed with neuronal tracing and was found to differ between species.

* Number after Hummel et al. 2011.
