# Supplementary material for: Position-dependent hearing in three species of bushcrickets (Tettigoniidae, Orthoptera)
Source: R Soc Open Sci. 2015 Jun 9;2(6):140473. doi: 10.1098/rsos.140473 (PMC4632538; doi:10.1098/rsos.140473)
Supplement: 3 Table with P values of hearing thresholds between males and females [file rsos140473supp3.docx]

**Supplementary information 3**

Summary of comparison between thresholds of females and males of the three tested species. Threshold data of all tested frequencies (13 frequencies each) were compared for five different positions of the loudspeaker.

Most hearing thresholds of females and males were not different. Only in SC, males had significantly higher thresholds for two frequencies (35 and 40 kHz) in the contralateral position (c80°, 0°) and three frequencies (20, 25 and 35 kHz) in the dorsal (zenith) position, respectively (** = P < 0.01). Given are summaries with P-value in brackets (n.s. = not significant).

Statistical test: Two-way ANOVA, with Bonferroni`s post test. For analysis sound pressure level data have been transferred to sound pressure. Analyses were performed with Prism4 (GraphPad Inc).

|  | ***Mecopoda elongata*** | ***Stilpnochlora couloniana*** | ***Ancylecha fenestrata*** |
| --- | --- | --- | --- |
| **N** | 6 males, 6 females | 6 males, 9 females | 4 males, 5 females |
| **Ipsilateral**  **(i80°, 0°)** | n.s. (0.7274) | n.s. (0.8468) | n.s. (0.3625) |
| **Contralateral**  **(c80°, 0°)** | n.s. (0.2349) | ** (0.0010) | n.s. (0.4458) |
| **Frontal**  **(0°, 0°)** | n.s. (0.9124) | n.s. (0.3023) | n.s. (0.5916) |
| **Dorsal**  **(zenith)** | n.s. (0.9996) | ** (0.0015) | n.s. (0.9014) |
| **Caudal**  **(180°, 0°)** | n.s. (0.9219) | n.s. (0.8206) | n.s. (0.9317) |
