# Supplementary material for: Position-dependent hearing in three species of bushcrickets (Tettigoniidae, Orthoptera)
Source: R Soc Open Sci. 2015 Jun 9;2(6):140473. doi: 10.1098/rsos.140473 (PMC4632538; doi:10.1098/rsos.140473)
Supplement: 4 Figure with characters of the calling songs of the experimental species [file rsos140473supp4.docx]

**Supplementary information 4**

Characteristics of the calling songs of the three investigated species of bushcrickets. Shown are single chirps with an oscillogram trace (top) and a spectrogram (bottom). Note the different scaling.

*M. elongata* males produce a calling song with a sequence of chirps. Each chirps (with about 400 ms duration) has a broadbanded spectrum of 7-70 kHz (recorded with an ultrasound microphone (Avisoft), connected to a digital recorder (Tascam HD-P2; 192 kHz sampling rate; temperature 24°C). Analysed with Adobe Audition and visualized with the program SoundRecognitionLab).

*S. couloniana* males produce a calling sounds with single chirps (about 140 ms duration) and with a peak at 7-12 kHz (recorded with an SR40 ¼ inch microphone (Earthworks), connected via an electronic interface expander (Akai EIE) to a computer; software: Adobe Audition, 96 kHz sample rate; temperature 22°C; visualized with the program SoundRecognitionLab).

*A. fenestrata* males produce a calling song with short single chirps (about 11 ms duration) and with a peak at 18-44 kHz (recorded by Dr. K. Kowalski with an ultrasound microphone (Avisoft), connected to a digital recorder (Tascam HD-P2; 192 kHz sampling rate; temperature 24°C). Analysed with Adobe Audition and visualized with the program SoundRecognitionLab).

| 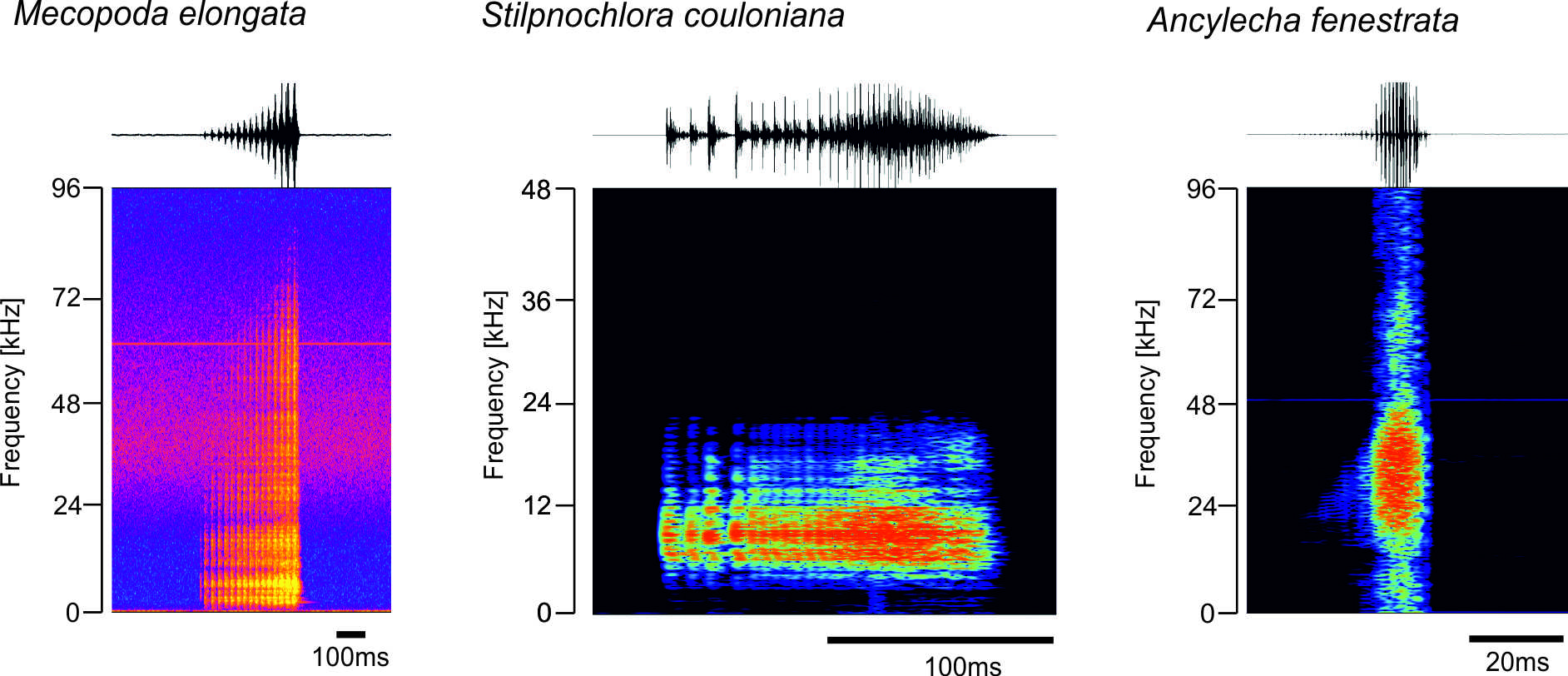 |
| --- |
